# Combined Computed Coronary Tomography Angiography and Transcatheter Aortic Valve Implantation (TAVI) Planning Computed Tomography Reliably Detects Relevant Coronary Artery Disease Pre-TAVI

**DOI:** 10.3390/jcm13164885

**Published:** 2024-08-19

**Authors:** Dominik Felbel, Christoph Buck, Natalie Riedel, Michael Paukovitsch, Tilman Stephan, Marvin Krohn-Grimberghe, Johannes Mörike, Birgid Gonska, Christoph Panknin, Christopher Kloth, Meinrad Beer, Wolfgang Rottbauer, Dominik Buckert

**Affiliations:** 1 Department of Cardiology, Angiology, Pneumology and Internal Intensive Care, Ulm University Heart Center, 89081 Ulm, Germany; 2 Department of Diagnostic and Interventional Radiology, Ulm University Medical Center, 89081 Ulm, Germany; 3 Scientific Collaborations Siemens Healthcare GmbH Erlangen, 91301 Forchheim, Germany

**Keywords:** coronary artery disease, coronary CT, cCTA, TAVI

## Abstract

**Background:** Before surgical or transcatheter aortic valve implantation (TAVI), coronary status evaluation is required. The role of combined computed coronary tomography angiography (cCTA) and TAVI planning CT in this context is not yet well elucidated. This study assessed whether relevant proximal coronary disease requiring coronary revascularization can be safely detected by combined cCTA and TAVI planning CT, including CT-derived fractional flow reserve (FFR) calculation in patients with severe aortic stenosis. **Methods:** This study analyzed patients with successful cCTA combined with TAVI planning CT using a 128-slice dual-source scanner. The detection via cCTA of relevant left main stem stenosis (>50%) or proximal coronary artery stenosis (>70%) was compared to invasive coronary angiography (ICA). **Results:** This study comprised 101 consecutive TAVI patients with a median age of 83 [77–86] years, a median STS score of 3.7 [2.4–6.1] and 54% of whom had known coronary artery disease. Of 15 patients with relevant coronary stenoses, 14 (93.3%) were detected with cCTA, while false positive results were found in 25 patients. Only in patients with previous percutaneous coronary stent implantation (PCI) were false positive rates (11/29) increased. In the subgroup without previous PCI, an improved classification performance of 87.5%, being mainly due to 11.1% false positive classifications, led to a negative predictive value of 98.5%. **Conclusions:** Combined cCTA and CT-FFR with TAVI planning CT via state-of-the-art scanners and protocols as a one-stop shop can replace routine ICA in patients prior to TAVI due to its safe detection of relevant coronary artery stenosis, although diagnostic performance of cCTA is only reduced in patients with coronary stents.

## 1. Introduction

According to the current European Society of Cardiology (ESC) and American College of Cardiology/American Heart Association (ACC/AHA) guidelines, coronary computed tomography angiography (cCTA) for coronary artery disease (CAD) evaluation in patients undergoing transcatheter aortic valve implantation (TAVI) is recommended in patients with a low pretest probability only [1,2]. This is mainly because of its excellent negative predictive value, while cCTA tends to overestimate CAD [1,2,3]. Consequently, in patients with aortic stenosis, who themselves already have a high prevalence for CAD, invasive coronary angiography (ICA) still represents the reference standard for interventional planning [1,2,4]. New-generation CT-scanners and the development of CT-derived fractional flow reserve (CT-FFR) have been shown to improve the diagnostic performance of cCTA in detecting CAD in these patients further [3,5,6,7].

The clinical benefit remains unclear, especially as to whether modern cCTA image acquisition and evaluation can safely detect relevant CAD—defined as proximal coronary artery > 70% or a left main stem stenosis > 50% during TAVI evaluation—when broadly applied. Therefore, our study aimed to assess whether cCTA can safely replace ICA in patients undergoing TAVI in an all-comer cohort.

## 2. Methods

### 2.1. Study Design and Cohort

This retrospective study included 130 consecutive patients undergoing combined cCTA and preprocedural TAVI planning CT between January and April 2021 at Ulm University Heart Center. State-of-the-art CT-based coronary artery evaluation was performed using a 128-slice dual-source CT via cCTA and machine-learning-based CT-FFR. CT-based detection of clinically relevant proximal coronary artery stenosis (angiographic stenosis > 70% of proximal coronary segments and >50% of the left main stem (LMS)) and the reference standard ICA were compared on a per patient analysis. The objective of this study was to determine if modern cCTA analysis can safely replace preprocedural ICA for the detection of clinically relevant coronary artery stenoses prior to TAVI. Therefore, a technical endpoint was defined as a successful complete readable cCTA acquisition. In patients reaching the technical endpoint, the clinical endpoint was achieved if the independent assessment of cCTA and invasive coronary angiography resulted in the same clinical conclusion, aiming at a clinical congruence endpoint. This study received no funding. The study was conducted according to the guidelines of the Declaration of Helsinki and approved by the Ethics Committee of Ulm University (No. 262/19). Informed consent was obtained from all subjects involved in the study.

### 2.2. Image Acquisition

Image acquisition was performed as described elsewhere [8]. In brief, the process involved a retrospectively ECG-gated helical scan of the heart, immediately followed by high-pitch scan of the torso utilizing a single bolus contrast medium using a third-generation dual-source MDCT scanner (Siemens Somatom Force dual source CT, Siemens Healthineers, Erlangen, Germany).

ECG-gated retrospective image acquisition was performed with an R-R-interval of 30–60%. Nitrates or beta blockers were not given. cCTAs were performed in line with the current guidelines of the Society of Cardiovascular Computed Tomography. Intravenous contrast media injection (Imeron 400, Bracco Imaging Germany) was applied in weight-adopted doses and injected at a flow rate of 4–6 mL/sec by antecubital vein. Timing bolus or bolus tracking was used, depending on the protocol.

### 2.3. CT-FFR, cCTA and ICA

CT-FFR was calculated from cCTA using on-site dedicated machine-learning-based research software (cFFR, version 3.5; Siemens Healthcare GmbH, Henkestr, Erlangen) [9]. Patients with previous percutaneous coronary intervention (PCI) and LMS were included, although the research software was not intended for these settings at first. An individual patient-specific model of the coronary artery tree was calculated for each patient. cCTA datasets were evaluated in consensus via visual analysis using thin-slab maximum intensity projections and curved multiplanar reconstructions. CT-FFR was determined using an on-site software prototype, as previously described [9]. First, all centerlines were automatically extracted by the software and had to be proofed by the radiologist. Second, a correction of the luminal contour had to be performed. Finally, a three-dimensional mesh representing the coronary artery tree was calculated. No side branches were evaluated. CT-FFR ≤ 0.8 was defined as significant stenosis. Relevant stenosis calculated from angiography CT (as a percentage) was defined as >50% in LMS and >70% in proximal coronary arteries. In order to display a “real-world” setting and to use the advances of both evaluation techniques, such as cCT reconstruction, patients with relevant stenosis in cCTA and/or CT-FFR, such as patients with relevant ostial stenosis visually detected in cCTA reconstruction (being unable to use CT-FFR), were classified as positive.

ICA was defined as the reference standard, available in all analyzed patients and evaluated independently by 2 experienced cardiologists. Angiographic stenosis > 70% of proximal coronary segments and >50% of the LMS were defined as relevant stenosis.

### 2.4. Statistical Analysis

The study cohort was divided into two groups based on the technical and the clinical endpoints. Results are presented as mean ± standard deviation, median with 25th–75th percentiles or proportions (%). The normality of the distribution of continuous variables was assessed using the Kolmogorov–Smirnov test. Normally distributed variables were compared by the Student’s *t*-test, while non-normally distributed were analyzed using the unpaired U-test. Categorial variables were compared using the chi-square test. Data availability for each variable are indicated in brackets. Variables potentially influencing the technical or clinical endpoint (*p* < 0.2) were further examined using univariate logistic regression analysis. The strength of associations with the endpoint was expressed by the odds ratio (OR) with 95% confidence intervals (CI). All statistical analyses were performed using IBM^®^ SPSS^®^ Statistics 28.0.1.0.

## 3. Results

**Figure 1:**
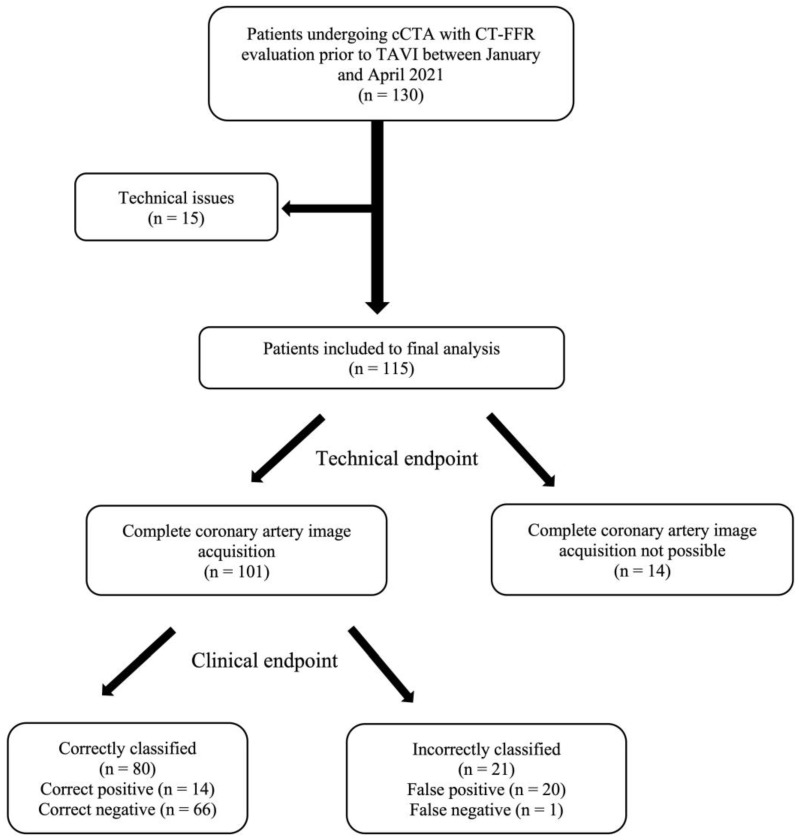
Flow chart of patient inclusion and stratification by the technical and clinical endpoint of 130 consecutive patients undergoing combined TAVI planning CT and coronary artery evaluation using cCTA and CT-FFR. Patients are stratified by the technical and the clinical endpoint. ICA was used as reference standard for correct or incorrect classification by cCTA. cCTA: computed coronary tomography angiography, CT-FFR: computed tomography derived functional flow reserve, ICA: invasive coronary angiography, TAVI: transcatheter aortic valve implantation.

Out of the initial 130 patients, 15 were excluded due to technical issues: coronary artery bypass graft (CABG) (*n* = 6), coronary angiography not available (*n* = 2), coronary anomalies (*n* = 5), datasets with software problems (*n* = 2). The remaining 115 patients comprised the study population, with a median age of 83 [77–86] years, a median STS score of 3.7 [2.4–6.1] and 48% were male and in 54% CAD was diagnosed from ICA. In these patients, the technical endpoint of achieving successful complete readable image acquisition was met in 101 (87.8%) patients. Among those who reached the technical endpoint, the clinical endpoint of correctly classifying clinically relevant proximal CAD was achieved in 80 (79.2%) patients. Of these, 14 (13.9%) were true positives and 66 (65.3%) were true negatives. In contrast, 20 (19.8%) patients were classified as false positives and 1 (0.9%) patient was a false negative (Figure 1).

Table 1 stratifies patients according to the technical endpoint. Significant differences were observed in the presence of chronic obstructive pulmonary disease (COPD) (5.9 vs. 28.6% in patients missing the technical endpoint; *p* = 0.005), Troponin T levels (median 22 [15–38] vs. 39 [24–57] ng/L; *p* = 0.024) and heart rate during image acquisition (median 74 [66–82] vs. 58 [51–71] bpm; *p* = 0.003). Univariate logistic regression identified COPD to significantly increase the risk for missing the technical endpoint (OR 6.33 [95%-CI 1.53–26.28]; *p* = 0.011), Table 2.

Table 3 compares patients according to the clinical endpoint. Patients achieving the clinical endpoint had significantly lower rates of known CAD (36.3 vs. 71.4% in the group that missed the clinical endpoint; *p* = 0.004) and prior PCI (21.3 vs. 57.1%; *p* = 0.001). Additionally, these patients were less frequently male (38.8 vs. 66.7%; *p* = 0.022) and had a significantly higher body mass index (BMI) (27.3 [24.2–31.1] vs. 24.5 [21.9–29.1] kg/m^2^; *p* = 0.036). Univariate logistic regression indicated that a higher BMI (OR 0.89 [95%-CI 0.80–0.99]; *p* = 0.047) was associated with an increased likelihood of reaching the clinical endpoint. Conversely, prior PCI (OR 4.94 [95%-CI 1.79–13.66], *p* = 0.002), male sex (OR 3.16 [1.15–8.70]; *p* = 0.026) and known CAD (OR 4.39 [95%-CI 1.54–12.57]; *p* = 0.006) were associated with a lower likelihood of achieving the clinical endpoint (Table 4).

After exclusion of patients with prior PCI, which had the highest impact on the clinical endpoint in logistic regression analysis, 63 (87.5%) patients were correctly classified, 8 patients (11.1%) were false positive and 1 patient (1.4%) was classified as a false negative.

**Figure 2:**
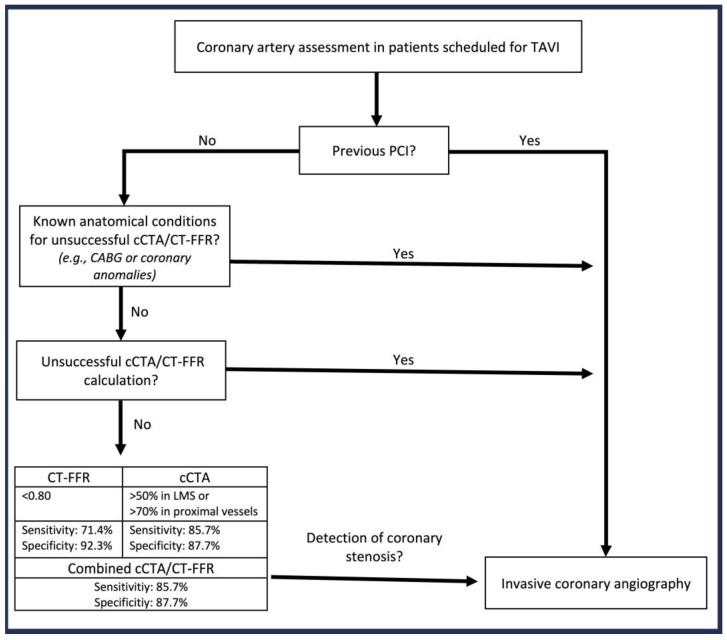
Patient selection algorithm for coronary artery assessment in TAVI patients. CABG = coronary artery bypass graft, cCTA = computed coronary tomography angiography, CT-FFR = computed tomography derived functional flow reserve, PCI = percutaneous coronary intervention, ICA = invasive coronary angiography, TAVI = transcatheter aortic valve implantation.

While sensitivity was higher with cCTA (93.3% in the whole cohort; 85.7% in patients without prior PCI), specificity was higher with CT-FFR (83.7% in the whole cohort; 92.3% in patients without prior PCI) (Supplements Table S1). Figure 2 outlines an algorithm for adequate patient selection to ensure safe CAD assessment using cCTA.

## 4. Discussion

This study is the first to identify predictors of successful assessment of clinically relevant CAD using state-of-the-art cCTA image acquisition and evaluation in the context of TAVI planning. By focusing on a patient-level analysis within a real-world setting, the main results of our research can be summarized as follows:

- Correct classification of relevant CAD was achieved in nearly 80% of patients.
- Prior PCI increases the likelihood of incorrect CAD assessment (OR 4.94, *p* = 0.002).
- Excluding patients with prior PCI increases correct classification to 88%, while misclassification was mainly driven by 11% false positives, leading to high patient safety.
- COPD is the only predictor of unsuccessful image acquisition (OR 6.3, *p* = 0.011).

Our findings confirm that cCTA tends to overestimate coronary artery stenosis, resulting in moderate specificity of 73%, as previously reported [3]. Contrarily, we demonstrate that using CT-FFR increases specificity, while sensitivity is slightly reduced compared to cCTA. These findings are in line with various studies such as data from the Prospective LongitudinAl Trial of FFRct: Outcome and Resource IMpacts (PLATFORM) and analyses of patients undergoing combined TAVI planning CT and cCTA [3,5,10].

However, the performance of cCTA and CT-FFR to detect CAD varies depending on the predefined cut-off values and the scanner type. In former studies, cCTA cut-off values range between ≥50 and >70%, CT-FFR between ≤0.80 and ≤0.75 and different generation CT-scanner with 64–320 slice CT were used [3,5,6,11,12]. Furthermore, there is variability across studies that either investigate the presence of CAD or aim at detecting clinically relevant CAD only [3,13]. While some studies aimed to identify CAD ≥ 50% in ICA, our study aimed to assess the diagnostic performance of CT to detect clinically relevant CAD, only defined as >70% in proximal vessels or >50% in LMS, according to current guidelines prior to heart valve intervention [1,2,3,5].

This study aimed to further improve the diagnostic performance of modern coronary CT prior to TAVI by adequate patient selection to safely replace ICA. Logistic regression revealed that coronary artery stent implantation impaired correct CAD classification, leading to a higher rate of false positives in cCTA. In prior studies, stent implantation was defined as an exclusion criterion due to its lacking suitability for CT-FFR [5]. Interestingly, even though the machine learning-based CT-FFR software used in our study is not validated for patients with stents, it still reduced the number of false positives, achieving a moderate specificity of 71%. However, stent evaluation using coronary CT is known to be challenging, especially in small size stents and calcified plaques [14,15].

Excluding patients with prior PCI significantly improved diagnostic performance, resulting in 88% correct classification for clinically relevant CAD, with less than 1% classified as false negatives. Consequently, in patients without prior PCI reaching the technical endpoint, cCTA already leads to acceptable results when compared to ICA offering a safe alternative to ICA. Based on these convincing results, we developed an algorithm to select the most suitable procedure for CAD diagnostics prior to TAVI (Figure 2). Additional radiation can be avoided in patients with prior stent implantation when considering the impaired diagnostic performance. However, machine-learning-based image reconstruction has been shown to improve image sharpness and reduced image noise in patients with coronary stents, which may offer safe coronary stent evaluation, even in small size stents, in the future [14]. Additionally, a recent study demonstrated that photon-counting CT provides high diagnostic accuracy, even in patients with severe coronary calcification or prior stent placement. However, with the use of ultrahigh-resolution cCTA, radiation dose was noticeably higher compared to conventional computed tomography [16].

As a precondition for adequate CAD assessment, a technical endpoint was defined as complete readable cCTA. COPD significantly increased the risk of unsuccessful image acquisition, primarily due to breath artifacts, explained by these patients’ inability to maintain a breath-hold maneuver. However, in patients with successful image acquisition, COPD did not impair the diagnostic performance of cCTA and consequently should not be used as general exclusion criterion for cCTA prior to TAVI. Interestingly, heart rate was observed to be significantly lower in patients with unsuccessful image acquisition. However, recent studies suggest that a heart rate above 80 beats per minute increases the risk of unsuccessful image acquisition, making this finding likely a by chance observation.

Current guidelines recommend cCTA for patients undergoing heart valve interventions with a low risk for CAD only [1,2]. This study demonstrates that adequate patient selection enhances safety, while offering the benefit of cCTA combined with TAVI planning CT in a larger proportion of patients. This approach also advances the concept of “fast-track TAVI” and the TAVI planning CT as a “one stop shop” offering complete outpatient TAVI planning in selected patients, thereby reducing hospital stay and associated risks and costs.

## 5. Limitations

The results of this study have to be interpreted with several limitations. The patient sample size is limited and further studies are needed to confirm these results. CT-FFR evaluation was performed with a machine-learning-based research software not intended for left main stem or stent evaluation. However, the promising results for stent evaluation suggest potential future applications in diagnostics.

## 6. Conclusions

Combined cCTA and CT-FFR is already able to safely detect clinically relevant CAD during TAVI planning CT. Therefore, routine invasive coronary angiography can be safely omitted in TAVI patients without prior PCI and in whom cCTA can be technically performed as part of the planning CT. Future software advancements may further advance the diagnostic performance for stent evaluation, especially using CT-FFR.

In clinical practice, ICA displays the reference standard for CAD assessment in patients scheduled for TAVI. However, cCTA using new-generation CT-scanners including CTA and CT-FFR evaluation safely detects clinically relevant proximal coronary artery stenosis according to current guidelines in selected patients. For patients without previous PCI in whom cCTA can be technically performed, ICA can safely be replaced by cCTA enabling complete outpatient TAVI planning.

## Figures and Tables

**Table 1:** Baseline characteristics and image acquisition of patients undergoing cCTA for TAVI planning stratified by the technical endpoint.

	Technical Endpoint Reached (*n* = 101)	Technical Endpoint Missed (*n* = 14)	*p*-Value
Age, years	82.8 [76.7–85.5] (101/101)	85.1 [77.6–87.9] (14/14)	0.130
Height, cm	165.0 [160.0–172.0] (101/101)	173.5 [159.0–176.0] (14/14)	0.164
Weight, kg	73 [65–88] (101/101)	82 [68–89] (14/14)	0.541
BMI, kg/m^2^	26.7 [23.8–30.5] (101/101)	27.9 [24.1–30.4] (14/14)	0.868
Male	45 (44.6%) (101/101)	10 (71.4%) (14/14)	0.059
Known coronary artery disease	44 (43.6%) (101/101)	7 (50.0%) (14/14)	0.650
Prior PCI	29 (28.7%) (101/101)	3 (21.4%) (14/14)	0.569
Arterial hypertension	87 (86.1%) (101/101)	13 (92.9%) (14/14)	0.484
Diabetes	28 (27.7%) (101/101)	3 (21.4%) (14/14)	0.619
Hyperlipidemia	61 (60.4%) (101/101)	10 (71.4%) (14/14)	0.426
ICD or pacemaker	4 (4.0%) (101/101)	2 (14.3%) (14/14)	0.103
COPD	6 (5.9%) (101/101)	4 (28.6%) (14/14)	**0.005**
Dialysis	1 (1.0%) (101/101)	0 (14/14)	0.708
eGFR, mL/min	59 ± 20 (101/101)	67 ± 21 (14/14)	0.164
CKD stage - 1 - 2 - 3 - 4 - 5	5 (5.0%) 41 (40.6%) 49 (48.5%) 5 (5.0%) 1 (1.0%)	1 (7.1%) 9 (64.3%) 4 (28.6%) 0 0	0.464
Troponin T, ng/L	22 [15–38] (101/101)	39 [24–57] (14/14)	**0.024**
NT-pro BNP pg/L	1493 [517–5135] (98/101)	2213 [741–4947] (14/14)	0.389
LVEF - >55% - 45–54% - 30–44% - <30%	65 (64.4%) 10 (9.9%) 8 (7.9%) 18 (17.8%) (101/101)	6 (42.9%) 2 (14.3%) 2 (14.3%) 4 (28.6%) (14/14)	0.482
Atrial fibrillation or flutter	49 (48.5%) (101/101)	8 (57.1%) (14/14)	0.545
Coronary CT-Angiography			
Heart rate during acquisition, bpm	74 [66–82] (94/101)	58 [51–71] (13/14)	**0.003**
mAs	7754 ± 2662 (101/101)	8470 ± 3266 (14/14)	0.499
DLP, mGycm	626 [404–715] (101/101)	661 [335–1048] (14/14)	0.521
Contrast, mL	76 [71–86] (101/101)	76 [71–86] (14/14)	0.619
Cardiac mass, g	188 [154–240] (101/101)	-	
Invasive Coronary Angiography			
Coronary artery disease none 1-vessel 2-vessel 3-vessel	48 (47.5%) 16 (15.8%) 12 (11.9%) 25 (24.8%) (101/101)	5 (35.7%) 1 (7.1%) 3 (21.4%) 5 (35.7%) (14/14)	0.489

BMI = body mass index, bpm = beats per minute, cCTA = computed coronary tomography angiography, COPD = chronic obstructive pulmonary disease, CKD = chronic kidney disease, DLP = dos length product, eGFR = estimated glomerular filtration rate, ICA = invasive coronary angiography, ICD = implantable cardioverter defibrillator, LVEF = left ventricular ejection fraction, mAs = milliampere-seconds, PCI = percutaneous coronary intervention, TAVI = transcatheter aortic valve implantation. *p*-values < 0.05 are presented bold. Data availability is displayed in brackets below for each variable.

**Table 2:** Logistic regression analysis to identify predictors for missing the technical endpoint.

Univariate Logistic Regression Analysis			
	Odds Ratio	95%-Confidence Interval	*p*-Value
Age, years	1.08	0.98–1.18	0.132
Height, cm	1.04	0.97–1.10	0.271
Male	3.11	0.92–10.58	0.069
ICD or pacemaker	4.04	0.67–24.46	0.128
COPD	6.33	1.53–26.28	**0.011**
eGFR, mL/min	1.02	0.99–1.05	0.166
Troponin T, ng/L	1.000	0.994–1.001	0.971

COPD = chronic obstructive pulmonary disease, eGFR = estimated glomerular filtration rate, ICD = implantable cardioverter defibrillator, *p*-values < 0.05 are presented in bold.

**Table 3:** Baseline characteristics and image acquisition of patients undergoing cCT-A for TAVI planning stratified by the clinical endpoint.

	Clinical Endpoint Reached (*n* = 80)	Clinical Endpoint Missed (*n* = 21)	*p*-Value
Age, years	81.6 [76.7–84.6] (80/80)	84.5 [77.9–88.9] (21/21)	0.067
Height, cm	165.3 ± 9.3 (80/80)	168.1 ± 8.2 (21/21)	0.216
Weight, kg	74.5 [65.0–88.0] (80/80)	67 [61.5–80.5] (21/21)	0.194
BMI, kg/m^2^	27.3 [24.2–31.1] (80/80)	24.5 [21.9–29.1] (21/21)	**0.036**
Male	31 (38.8%) (80/80)	14 (66.7%) (21/21)	**0.022**
Known coronary artery disease	29 (36.3%) (80/80)	15 (71.4%) (21/21)	**0.004**
Prior PCI	17 (21.3%) (80/80)	12 (57.1%) (21/21)	**0.001**
Arterial hypertension	69 (86.3%) (80/80)	18 (85.7%) (21/21)	0.950
Diabetes	23 (28.7%) (80/80)	5 (23.8%) (21/21)	0.653
Hyperlipidemia	49 (61.3%) (80/80)	12 (57.1%) (21/21)	0.732
ICD or pacemaker	3 (3.8%) (80/80)	1 (4.8%) (21/21)	0.832
COPD	5 (6.3%) (80/80)	1 (4.8%) (21/21)	0.797
Dialysis	0 (80/80)	1 (4.8%) (21/21)	0.050
eGFR, mL/min	60 ± 20 (80/80)	54 ± 20 (21/21)	0.250
CKD stage - 1 - 2 - 3 - 4 - 5	4 (5.0%) 34 (42.5%) 38 (47.5%) 4 (5.0%) 0 (80/80)	1 (4.8%) 7 (33.3%) 11 (52.4%) 1 (4.8%) 1 (4.8%) (21/21)	0.375
Troponin T, ng/L	22 [15–73] (80/80)	29 [16–45] (21/21)	0.364
NT-pro BNP pg/L	1328 [536–4734] (77/80)	1587 [459–7993] (21/21)	0.396
LVEF - >55% - 45–54% - 30–44% - <30%	53 (66.3%) 6 (7.5%) 7 (8.8%) 14 (17.5%) (80/80)	12 (57.1%) 4 (19.0%) 1 (4.8%) 4 (19.0%) (21/21)	0.422
Atrial fibrillation or flutter	36 (45.0%) (80/80)	13 (61.9%) (21/21)	0.168
Coronary CT-Angiography			
Heart rate during acquisition, bpm	74 ± 12 (75/80)	73 ± 13 (19/21)	0.764
mAS	7932 [5968–9373] (80/80)	7593 [5360–9009] (21/21)	0.547
DLP, mGycm	640 [414–811] (80/80)	521 [299–677] (21/21)	0.098
Contrast, mL	76 [71–86] (80/80)	71 [71–76] (21/21)	**0.007**
Cardiac mass, g	195 [167–246] (80/80)	173 [137–215] (21/21)	0.098
Invasive Coronary Angiography			
Coronary artery disease none 1-vessel 2-vessel 3-vessel	45 (56.3%) 10 (12.5%) 7 (8.8%) 18 (22.5%) (80/80)	3 (14.3%) 6 (28.6%) 5 (23.8%) 7 (33.3%) (21/21)	**0.005**

BMI = body mass index, bpm = beats per minute, cCTA = computed coronary tomography angiography, COPD = chronic obstructive pulmonary disease, CKD = chronic kidney disease, DLP = dose length product, eGFR = estimated glomerular filtration rate, ICA = invasive coronary angiography, ICD = implantable cardioverter defibrillator, LVEF = left ventricular ejection fraction, mAs = milliampere-seconds, PCI = percutaneous coronary intervention, TAVI = transcatheter aortic valve implantation. *p*-values < 0.05 are presented bold. Data availability is displayed in brackets below for each variable.

**Table 4:** Logistic regression analysis to identify predictors for missing the clinical endpoint.

Univariate Logistic Regression Analysis			
	Odds Ratio	95%-Confidence Interval	*p*-Value
Age, years	1.07	0.99–1.16	0.104
Weight, kg	0.98	0.95–1.01	0.217
BMI, kg/m^2^	0.89	0.80–0.99	**0.047**
Prior PCI	4.94	1.79–13.66	**0.002**
Atrial fibrillation or flutter	1.99	0.74–5.32	0.172
Male	3.16	1.15–8.70	**0.026**
Known coronary artery disease	4.39	1.54–12.57	**0.006**
Cardiac mass, g	0.99	0.99–1.00	0.133

BMI = body mass index, PCI = percutaneous coronary intervention. *p*-values < 0.05 are presented bold. Data availability is displayed in brackets below for each variable.

## Data Availability

Study data are available from the corresponding author upon reasonable request.

## Supplementary Materials

The following supporting information can be downloaded at: https://www.mdpi.com/article/10.3390/jcm13164885/s1, Table S1: Classification of patients with relevant stenosis and false positive classified patients stratified by cCTA or CT-FFR assessment.

## Abbreviations

BMI	body mass index
CABG	coronary artery bypass graft
CAD	coronary artery disease
CT	computed tomography
cCTA	computed coronary tomography angiography
CT-FFR	computed tomography derived functional flow reserve
COPD	chronic obstructive pulmonary disease
ECG	Electrocardiogram
LMS	left main stem
MDCT	multidetector computed tomography
ICA	invasive coronary angiography
OR	odds ratio
PCI	percutaneous coronary intervention
STS	society of thoracic surgeons
TAVI	transcatheter aortic valve implantation
