# Supplementary material for: Combined Computed Coronary Tomography Angiography and Transcatheter Aortic Valve Implantation (TAVI) Planning Computed Tomography Reliably Detects Relevant Coronary Artery Disease Pre-TAVI
Source: J Clin Med. 2024 Aug 19;13(16):4885. doi: 10.3390/jcm13164885 (PMC11355140; doi:10.3390/jcm13164885)
Supplement: Supplementary file 1 [file jcm-13-04885-s001.zip › jcm-3110558-supplementary.pdf]

Supplementary Material

Supplemental table S1 Classification of patients with relevant stenosis and false positive classified patients stratified by cCTA or CT-FFR assessment

	cCTA	CT-FFR
Whole cohort (n = 101)		
Patients with relevant stenosis in ICA (n = 15)	14/15 (93.3%) Sensitivity 93.3%	11/15 (73.3%) Sensitivity 73.3%
False positive classified patients in cCT or CT-FFR (n = 25)	25/25 (0%) Specificity 70.9%	11/25 (44.0%) Specificity 83.7%
Patients without prior PCI (n = 72)		
Patients with relevant stenosis in ICA (n = 7)	6/7 (85.7%) Sensitivity 85.7%	5/7 (71.4%) Sensitivity 71.4%
False positive classified patients in cCT or CT-FFR (n = 8)	8/8 (0%) Specificity (87.7%)	5/8 (37.5%) Specificity (92.3%)
Patients with prior PCI (n = 29)		
Patients with relevant stenosis in ICA (n = 8)	8/8 (100%) Sensitivity 100%	4/8 (50%) Sensitivity 50.0%
False positive classified patients in cCT or CT-FFR (n = 11)	11/11 (100%) Specificity 47.6%	6/11 (54.6%) Specificity 71.4%

cCTA: computed coronary tomography angiography, CT-FFR: computed tomography derived functional flow reserve, ICA: invasive coronary angiography, PCI: percutaneous coronary intervention
